# Cases Admitted under the Care of the Surgeon of the Finsbury Dispensary, St. John's Square, Clerkenwell, from the 10th of June to the 10th of August, 1802

**Published:** 1802-09-01

**Authors:** J. Ricards

**Affiliations:** No. 68, Hatton Street


*Cases admitted under the Care of the Surgeon of the Finsbury Dispensary, St. John's Square, Clerkenwell, from the* 10*th of June to the* 10*th of Auguft*, 1802.

[The former Report having been intended for a publication not exclufively of a Medical nature, the names of the difeafes were inferted in Englifh; thofe terms being preferred, which, it was thought, would be moft eafily underftood by the general reader: But the Report being transferred to the Medical and Phyfical Journal, it has been fince thought more agreeable to the nature of that work, to give the lift of difeafes a more fcientific arrangement ment and appearance, by rendering them according to their claſſical appellation.]

| | | | |
|---|---|---|---|
| Phlegmone Artuum - - | 4 | Vulnera Capitis - - - - | 1 |
| ——— Articulorum - | 3 | Contuſiones - - - - - | 13 |
| ——— Teſtis - - - | 1 | Combuſtura - - - - - - | 4 |
| Ophthalmia - - - - - - | 1 | Lues - - - - - - - - - | 3 |
| Eryſipelas - - - - - - | 2 | Gonorrhœa virul. - - - | 2 |
| Abſceſſus Artuum - - - | 8 | ——— pura - - - | 1 |
| ——— Maxillæ - - - | 3 | Scrophula - - - - - - | 5 |
| ——— Ani - - - - | 2 | Pſora - - - - - - - - | 2 |
| ——— Lateris - - - | 1 | Strictura Urethræ - - - | 1 |
| ——— Labii pudendi - | 1 | Verrucæ - - - - - - - | 1 |
| ——— Pſoas - - - - | 2 | Tinea - - - - - - - - - | 1 |
| Mortificatio Artuum - - | 2 | Herpes - - - - - - - | 1 |
| Ulcera Faciei - - - - - | 2 | Varices - - - - - - - | 5 |
| ——— Artuum - - - | 25 | Exoſtoſis - - - - - - - | 1 |
| Fiſtula Scroti - - - - - | 1 | Spina incurvata - - - - | 1 |
| Contractio Articuli - - | 1 | Dyſecœa - - - - - - - | 1 |
| Leucoma - - - - - - | 1 | Hernia - - - - - - - | 2 |
| Fractura Ulnæ - - - - - | 1 | Ganglion - - - - - - | 1 |
| Vulnera Artuum - - - | 4 | | |

The very great and ſudden alteration which has taken place in the temperature of the atmoſphere, during the latter part of the time which the preſent Report embraces, has cauſed a conſiderable alteration in the aſpect of diſeaſes. The phyſicians of the metropolis have, doubtleſs, obſerved a viciſſitude in the nature of thoſe maladies which peculiarly fall under their cognizance: The diſeaſes of ſurgery have, in a proportionate degree, demonſtrated the influence which external cauſes exert over them.

Either extreme in the ſcale of the temperature of the atmoſphere muſt produce ſuch effects on a living body, as muſt, more or leſs, according to the exiſting ſtate of health and vigour, derange the natural, vital, or animal functions. This more eſpecially happens, when theſe extremes alternate ſuddenly with each other.

In every claſs of ſociety they muſt manifeſt their agency in ſome degree; but it is in the lower order of mankind that their effects are more particularly experienced, when the means cannot be enjoyed of guarding againſt the primary influence of theſe circumſtances, or of obviating the ſecondary cauſes which reſult therefrom, and which, in an increaſed ratio as the cauſes are multiplied, tend to induce diſeaſe, or, where diſeaſe does already exiſt, give birth to ſymptoms and appearances of an untoward nature, which otherwiſe might never have occurred.

There

There is no difeafe, which, in a more remarkable manner manifefts the alteration produced by a change of temperature, than the ulcers of weak, relaxed, and irritable habits. An ulcer in fuch a conftitution, of which there are very many among the poorer ranks of the community, upon the fudden acceffion of extremely warm weather, although before the change its appearance was kindly, very foon changes its afpect; the furface puts on a brown, unhealthy hue; ulceration extends at its edges, and oftentimes a fore of fcarcely half an inch in diameter will in a few days fpread round the limb. Neither are thefe changes of appearances to be wondered at, as no part partakes fo much of, and points out with more accuracy, the general health of the body, than an ulcerated furface. During the firft attack of a fever, we obferve the granulations of an ulcer become pale, and fhrink beneath the furface: as the fever advances, efpecially in fevers of debility, which are the moft common; indeed, I believe I may fay the only fever of the metropolis, the furface becomes in a foul and floughy ftate, which is in fact, mortification of the granulations: fo extreme heat, producing in relaxed and irritable habits indirect debility from the great exhauftion which is the natural confequence of it, is accompanied with an equally inactive ftate of the veffels of the ulcer, the procefs of fuch granulation is ftopped, and the parts which have already healed, very often fly out again, as it is termed. As very flight exciting caufes will produce an ulceration in newly formed fkin, or in fkin which has been produced to cover any previoufly ulcerated furface, as navy furgeons are accuftomed to fee the cicatrices of old ulcers fly out on an attack of fcurvy, or other difeafes of great debility. But independently of the effect which extreme heat muft have on the ulcer by means of the conftitution, it muft manifeft its local action likewife very injurioufly, for heat tends very much to expedite the procefs of putrefaction in animal fluids, and this effect it has in a very forcible manner in the fluids fecreted from an ulcer, as our fenfes moft powerfully evince to us, on approaching a large ulcer in the prefent ftate of the weather: this, by being confined, even for a few hours, muft excite on the furface of the ulcer an action of an unhealthy nature, and more efpecially when attended with a degree of uncleanlinefs, which in a larger proportion of the lower order of people, appears to be innate, and never to be forfaken.

I have obferved in a few cafes during the laft week of the prefent Report, that extenfive gangrene has fucceeded a very apparently trivial accident: in one cafe a fphacelus of fix inches in length, occurred merely in confequence of fcratching a pimple from the leg.

As

As we fee the effects which extreme heat manifefts in ulcers in fome conftitutions, it becomes our duty to endeavour to prevent thefe effects, where we fufpect fuch a ftate of conftitution to exift: To prevent debility from excefs of heat in fuch cafes, I find moft benefit refults from the mineral acids, as they tend to invigorate the fyftem without adding any increafe of ftimulus. To guard againft the ill effects of heat locally, the applications which I prefer are cold lotions; it fignifies little what are the compofition of thefe; the more fimple perhaps the better. I have feen the greateft poffible advantage refult (in cafes wherein the application could be attended to) from the conftant repetition of cold water only to the ulcer, which fhould be renewed as often as the fenfe of cold diminifhes: this requires, however, that a perfon's whole time fhould be employed in the management of his difeafe, which can feldom happen, except in cafes in which their peculiar nature renders confinement indifpenfable.

The advantage of cold applications in warm weather, I had an opportunity of obferving lately in a cafe of extenfive gangrene, in which the ufual application of beer-ground poultices, or indeed of any poultice, gave the moft excruciating pain, which yielded to the above treatment of cold lotions, often renewed, under which the flough feparated, and the fore is now in a promifing ftate.

Among the difeafes of furgery which have rarely been cured comparatively to the frequency of their occurrence, may be reckoned the pfoas abfcefs. Practitioners have, however, in feveral inftances, lately, performed a cure by the method pointed out by the judicious Mr. Abernethy, which confifts of evacuating the contents of the abfcefs by fmall punctures, frequently repeated, whereby the fides of the fac gradually collapfe and adhere: where this operation is not performed, and the abfcefs burfts fpontaneoufly, the patient feldom recovers; for, after a few days, fymptoms of irritation come on, which prefently terminate in hectic and death.

That this event, however, does not always follow the fpontaneous burfting of a pfoas abfcefs, is evident from a cafe which occurred lately at this charity: The patient had three years previoufly fuffered an injury in his loins by carrying a heavy weight; an abfcefs formed, and was opened in the loins, which healed, and he was then enabled to purfue his ufual occupation, but ftill was afflicted with a conftant pain at the lower part of his back. Several months after a tumour formed in the groin, which continued to enlarge, till at length it burft in two places, and difcharged a confiderable quantity of purulent matter, at which time he was admitted at the Difpenfary as a patient. On the the firft view of him, I did not expect that he would furvive long, as the difcharge was prodigious, and fymptoms of irritation, profufe fweating, great debility and lofs of appetite, had begun to take place: but, contrary to my expectations, by the free employment of fuch a plan as was adapted to invigorate the powers of the conftitution, and by fuitable applications to the part itfelf, he gradually recovered from thefe fymptoms; the difcharge ceafed from the cavity of the abfcefs, and he is now able to follow his cuftomary employment, in better health than he has enjoyed for feveral years.

*No.* 68, *Hatton Street.* J. RICARDS.

